# Supplementary material for: Learning curve and surgical outcome of robotic assisted colorectal surgery with ERAS program
Source: Sci Rep. 2022 Nov 29;12:20566. doi: 10.1038/s41598-022-24665-w (PMC9709162; doi:10.1038/s41598-022-24665-w)
Supplement: Supplementary file 1 — Supplementary Information 1. [file 41598_2022_24665_MOESM1_ESM.zip › LSC Raw data-Ñ[▒K/121/O_ñw╝╨░O▒Kñσ.pdf]

正式摘要(電子簽章：000000212342727)

請選擇...reporter

臺中榮民總醫院

Operation Note

[redacted]

- 手術日期：2021/03/15
- 手術醫師：VS 蔣鋒帆，CR 林陳彥，Fellow 黃俋霖，R 劉佳樺，R 楊立宇
- Anesthesia：GE
- Position：Lithotomy
- Aseptic agents：Betadine Aqua，Zephnan

Diagnosis

- Pre-operative diagnosis :S-colon tumor with liver metastasis and tumor bleeding status
- Post-operative diagnosis : s/p laparoscopic anterior resection

Surgical Indication

- For relieving the partial obstruction and bleeding condition

Operation

- 1. Laparoscopic anterior resection
  2. Intraoperative colonoscopy : (-)
  3. High ligation (+) : At IMA root
  4. EndoGIA (60-4.1) X1 + EEA (29 mm) were used
  5. Proximal cut end : 5 cm, distal cut end : 2 cm
  6. One 12 Fr. penrose drainage tube was placed into the pelvic cavity from the LLQ of the abdomen
  7. ADHESION BARRIER FILM was placed below the central abdominal wound.
  8. The colon was not taken down from the splenic flexure site
  9. The injured middle T-colon was repaired with Gambee Suture via 3-O Vicryl, then the outer layer was re-inforced by interrupted 3-0 silk Lembert sutures.

Finding

- 1. The ulcerative tumor, at least 5 X 5 cm with partial obstruction was presented.
  2. While entering the abdomen via the scopy, bloody ascites was presented over the pelvic area, S-colon area.
  3. The S-colon was moderately attached to the nearby abdominal wall, may be inflammation related.
  4. Multiple metastasis tumors could be easily seen over the liver surface. Suspicious, tumor ruptured like pattern was identified over the lower edge of the S3-S4. It was compressed with SurgiSeal, then with Surgicel fibrillar.
  5. Multiple LNs could be palpated over the IMA area, which was compatible with the abd CT findings.
  6. The injury of the middle T-colon was identified during the operation. It may be related to tumor adhesion or iatrogenic reason.

Procedure

- 1. General endotracheal anesthesia was induced and then the patient was placed in modified Trendelenburg lithotomy position.
  Preoperative antibiotics were given. A Foley catheter and a nasogastric tube were placed. The abdomen was prepped and draped in standard sterile fashion.
  2. A small vertical incision was made right next to the umbilicus and a 12 mm trocar was inserted into the peritoneal cavity by open method. Peumoperitoneum was established with CO2 inflation. Laparoscopy was inserted and then another 12 mm trocar was inserted over RLQ. Extra three 5 mm

- trochars were inserted over RUQ, LUQ and LLQ of abdomen. The operative findings were mentioned as above.
  3. The small bowel was inspected and retracted to the right by grasper. The sigmoid colon was then held by grasper and the peritoneum overlying the mesentery was then scored with electrocautery. The inferior mesentery artery was identified, ligated below the left colic artery with endoclips, and transected, so was inferior mesentery vein. Dissection was done above the Gerota's fascia and toward the peritoneal attachments.
  4. However, a small penetration wound was noted over the middle T-colon area, which was favored to be instrument induced laceration or tumor adhesion related.
  5. The midline wound was extended first and the T-colon was taken out of the abdomen for examination. It was repaired with Gambee Suture via 3-O Vicryl, then the outer layer was re-inforced by interrupted 3-0 silk Lembert sutures.
  6. We put the colon back into abdomen and then proceeded our AR operation.
  7. The arcade vessels to the sigmoid colon were also divided. Distal descending colon and upper rectum were mobilized. One staple of EndoGIA (60-4.1) was then used to divide the rectum.
  8. The small bowel was checked thoroughly again and no other suspicious injury sites were found.
  9. Then, The proximal colon was then taken out from midline extended wound for carefully inspection, and to make sure it has an excellent blood supply. The mesenteric fat was gently cleared from the terminal 2-3 mm of the bowel ends.
  10. The field was isolated with moist pad and the bowel opened and sized. It was determined that a 29 mm circular stapler could be accommodated. A pursestring suture of 2-0 prolene was placed. The tumor containing tissue was removed. The anvil was inserted, and then the pursestring suture was tied. The colon was then put back to the abdominal cavity.
  11. The anal canal was irrigated with distil water and then the circular stapler was introduced through the anus and pierced through the mid-portion of the stapler line.
  12. The proximal bowel with anvil in place was engaged with the circular stapler and the stapler was then fired. Two complete donuts were obtained and the distal one was submitted to pathology for margin confirmation.
  13. The anastomosis was tested for leaks with irrigation solution and air introduced through the rectum and was found to be patent and intact.
  14. The possible bleeder was then checked thoroughly.
  15. A 12 Fr. Penrose drainage tube was inserted from the LLQ 5mm trochar site into the anastomosis area (Pelvic).
  16. ADHESION BARRIER FILM was placed below the central abdominal wound.
  17. The fascia of midline wound was closed with interrupted sutures of Num. 1 Vicryl. The fascia of the 12 mm trochar was then closed with 1-0 Vicryl.
  18. Skin were closed with interrupted 3-0 nylon..
  19. The patient tolerated the procedure well and was brought to the POR in stable condition.
- Estimated blood loss: 300 c.c
- 器械數量: 正確
- 紗布數量: 正確

Image

- PAINT
  - abdomen

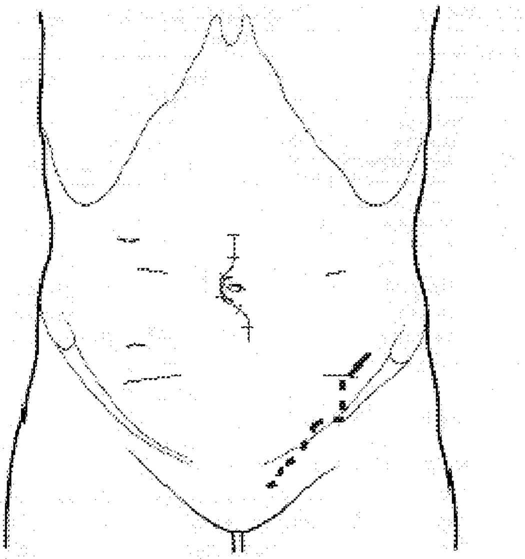

Blue line : Penrose drainage tube

- colon & rectum

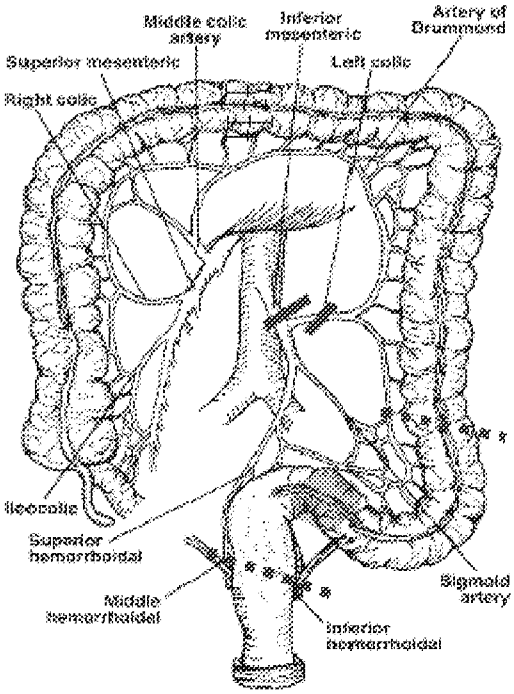

- The S-colon

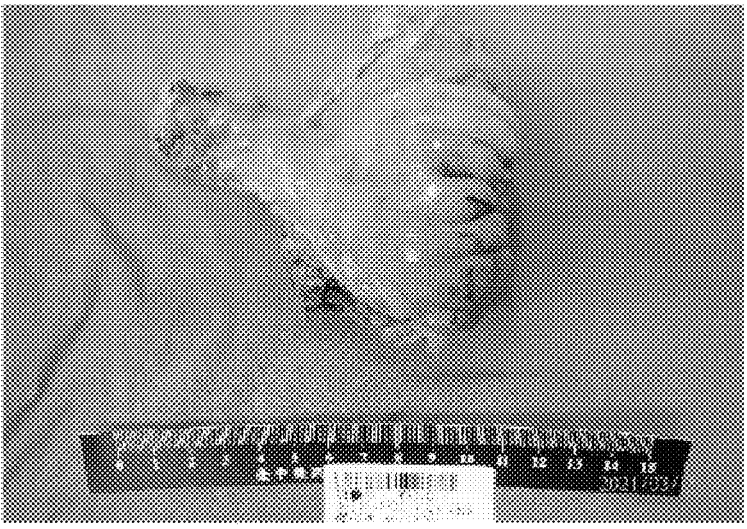

The tumor

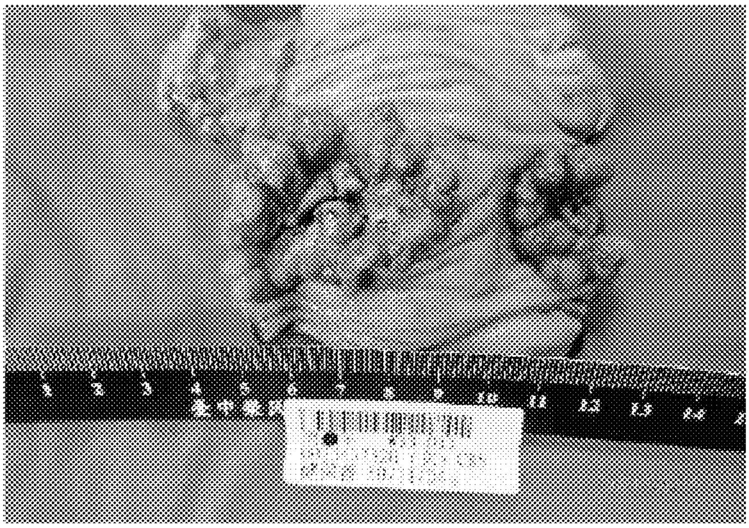

主任 2062F蔣鋒帆 / 醫師 2301H林陳彥

正式摘要(電子簽章：**000000212342727)**

請選擇...reporter

doctorRank:B
